# The effect of temperature on development and behaviour of relict leopard frog tadpoles

**DOI:** 10.1093/conphys/cow075

**Published:** 2017-02-14

**Authors:** Jeffrey A. Goldstein, Karin von Seckendorr Hoff, Stanley D. Hillyard

**Affiliations:** 1 Death Valley National Park, Pahrump Office, NV 89048, USA; 2 School of Life Sciences and School of Dental Medicine, University of Nevada, Las Vegas, Las Vegas, NV 89154, USA

**Keywords:** Amphibian, growth, larvae, preference, reintroduction, thermal, gradient

## Abstract

Relict leopard frog (*Rana* [*Lithobates*] *onca*) tadpoles were obtained shortly after hatching at Gosner stage 25 and raised in aquaria maintained at 15, 20, 25, 30 and 35°C. Development was arrested in the 15°C group, and survivorship declined to 64% after 191 days. However, 80% of the surviving larvae remained alive after the temperature was increased to 25°C. Of these, 96% reached metamorphosis. Survivorship of the 20, 25 and 30°C acclimation groups was 82, 94 and 66%, respectively, whereas none survived at 35°C. Time to metamorphosis was significantly shorter for the 25°C group (67 ± 1 days), followed by the 30°C (98 ± 2 days) and 20°C (264 ± 7 days) groups. A linear 66 cm thermal gradient was used to identify temperature ranges selected by tadpoles in the different acclimation groups. Five 10°C gradients (10–20, 15–25, 20–30, 25–35 and 30–40°C) were used, and time spent in the cooler, middle and warmer thirds of the gradient was compared for 10 individuals from each acclimation group. In the coolest gradient, tadpoles from all acclimation groups selected the warmer third (>17°C) of the gradient. In the warmer gradients, tadpoles from the 20 and 25°C acclimation groups selected temperatures <29°C, while those from the 30°C acclimation group selected temperatures <33°C. Maximal burst speed for all groups was greater at experimental temperatures of 25 than 15°C. Efforts to reintroduce this species to its historical range should select habitats where water temperatures between 25 and 30°C are available during the post-hatching period.

## Teaser

Relict leopard frogs, *Rana* [*Lithobates*] *onca*, once thought extinct, are confined to five known warm spring habitats along the Colorado River in Nevada. We evaluated the effects of temperature on developmental time, survival to metamorphosis and body mass at metamorphosis of captive-bred tadpoles. Highest survival and shortest time to metamorphosis were obtained at temperatures between 25 and 30°C, indicating that temperature is an important consideration for reintroduction site selection.

## Introduction

*Rana* [*Lithobates*] *onca* is part of the *Rana* [*Lithobates*] *pipiens* (leopard frog) complex, found from the northern half of Central America to Canada in North America (Stebbins and Cohen, 1995). Recent phylogenetic analyses (Crother, 2008) classified new world *Rana* species as *Lithobates*. More recent analyses (Yuan *et al*., 2016) have restored *Rana* as the appropriate generic classification, and we use it in the present study. However, it should be noted that at the time of this writing *Lithobates* is the currently accepted genus by the International Union for the Conservation of Nature (www.iucn.org) and the Society for the Study of Amphibians and Reptiles (Crother, 2008). *Rana pipiens* was once thought to be a single species complex, which has since been split up into 33 species in three subgroups, with *R. onca* and *R. yavapiensis* in a distinct clade (Jaeger *et al*., 2001; Hillis and Wilcox, 2005). When first described by Cope (1875), *R. onca* were commonly found around thermally influenced springs along the Virgin River drainage from Hurricane, Utah to the convergence with the Colorado River, into Black Canyon in Nevada (Wright and Wright, 1949; Jaeger *et al*., 2001; Bradford *et al*., 2004, 2005). At the time of the present study, there were only five remaining native sites, two located near the Overton arm of Lake Mead (Blue Point and Rogers Spring) and three located within Black Canyon just south of the Hoover Dam (Boy Scout Canyon, Salt Cedar Canyon, and Bighorn Sheep Spring; Jaeger *et al*., 2001). Water temperatures at the source of these springs exceed 30°C (Moran *et al*., 2015). As the spring outflow moves further down the drainage, water temperature is influenced by ambient temperature and varies seasonally from ~15°C during the winter months to >30°C in the summer (field observations). This provides a range of thermal environments that *R. onca*, which breed in the spring and winter months, can select for oviposition. However, the tadpoles may experience higher thermal conditions when development extends into the summer months.

Conservation efforts have been directed at captive breeding and reintroduction into once native habitats. Successful re-establishment of these populations requires continued reproduction in the face of multiple environmental variables (Tarszisz *et al*., 2014). The present study was initiated to isolate the effect of a single variable, water temperature between 15 and 35°C, on development of *R. onca* tadpoles. This range includes the temperatures that Pohlmann *et al*. (1998) documented for springs in the region where populations have historically been described. The following three criteria were observed: (i) survival to metamorphosis; (ii) time required to reach metamorphosis; and (iii) body mass at metamorphosis. In conjunction with the developmental study, tadpoles raised at each of the experimental temperatures were placed in thermal gradients to determine whether developmental temperature influenced thermal selection. Finally, burst speed was evaluated to determine whether physiological performance was affected by developmental temperature.

## Material and methods

### Collection and maintenance

Multiple *R. onca* egg masses that had been deposited in 17°C water were collected from Bighorn Sheep Spring, NV, USA on 21 January 2005 (seasonal spring source temperature, discharge rate and water composition of Bighorn Sheep Spring are available from Moran *et al*., 2015). Eggs were collected by the US National Park Service (permit no. S24409) by gently separating each egg mass in half, with the portion not attached to the vegetation placed in an insulated plastic container for transport, on the same day, to the Lake Mead National Recreation area facility in Boulder City, NV, USA. The eggs were housed at 21°C in 454 litre (120 gallon) aquaria, where they hatched on 25 January 2005. On 3 February 2005, 238 tadpoles were obtained from the US National Park Service at developmental stage 25 (Gosner, 1960), with an average body mass of 37 ± 20 mg (range 25–80 mg). Reported values are expressed as the mean ± 1 SEM unless otherwise indicated. Tadpoles were randomly divided into five groups ranging from 46 to 50 individuals. Each group was placed in a 114 litre (10 gallon) aquarium filled with dechlorinated Las Vegas tap water, at 22°C. The tadpole density was limited to 50 individuals per tank to reduce the effects of restricted space (John and Fusaro, 1981) or overcrowding (Martínez *et al*., 1996; Browne *et al*., 2003) on developmental rate and mass. Water in the aquaria was continuously filtered, aerated, and treated with both Stress Coat (Aquarium Pharmaceuticals, Inc., Chalfont, PA, USA) and Cycle Biological Aquarium Supplement (Rolf C. Hagen Corp., Mansfield, MA, USA) added as per product recommendations. Each aquarium contained gravel covering approximately one-third of the aquarium bottom, with an under-gravel biological sponge filter and synthetic vegetation. Water temperature was maintained with either a ViaAqua (Commodity Axis Inc., Camarillo, CA, USA) titanium tube electric aquarium heater (VA200T) or an AquaChill Industries Ltd (Vernon, BC, Canada) cooling unit (model no. AE3414YA). A plastic shelf was placed just below the water level for metamorphosing larvae to rest. Water temperature was adjusted by 1°C per day until acclimation temperatures of 15, 20, 25, 30 and 35°C were reached. Tadpoles were fed frozen spinach *ad libitum* to avoid the effects of dietary restriction (Merilä *et al*., 2004), and hardboiled egg albumen was added as a source of protein twice a week as per US National Park Service husbandry protocol. High water quality was maintained by exchanging a quarter of the water twice a week. All experimental procedures were approved by the University of Nevada, Las Vegas Institutional Animal Use and Care Committee (protocol R-701-0204-187).

### Analyses of development

Survivorship, developmental stage and mass were recorded two to three times per week until the initiation of metamorphosis (Gosner stage 42). Staging occurred by placing tadpoles individually on a moistened laboratory tissue to view under a stereoscope. All tadpoles for each acclimation group were weighed to the nearest 0.01 g.

### Thermal preference

A linear thermal gradient system (Sable Systems Inc., Las Vegas, NV, USA), was developed, with a 2-cm-thick aluminum floor (66 cm long × 6 cm wide) and Plexiglass sides. The aluminum floor, which served as a thermal conductor to establish and maintain a linear gradient, was connected on each end to Peltier heating/cooling units capable of establishing a 10°C gradient along the length of the chamber. The gradient chamber was filled ~2 cm deep with water obtained from the aquarium associated with the tadpole being tested. The gradient was verified with three thermocouples, one located 1 cm from either end and one in the centre, with the tip of the thermocouples ~3 mm above the aluminum floor.

The location of the tadpole was recorded every second by 64 LED sensors located 1 cm apart and 1 cm above the bottom of the gradient. When the LED beam was disrupted, the location was transmitted to a Linear Activity Detector (LAD; Sable Systems Inc., Las Vegas, NV, USA) and sent to a computer equipped with the LAD scan utility software that accumulated the sensor signals during a given trial. To prevent interference from external light sources and to reduce visual cues, a cover was placed above the gradient during experimental trials.

Thermal selection was evaluated with a subset of 10 individuals from the acclimation groups using the following five temperature ranges: 10–20, 15–25, 20–30, 25–35 and 30–40°C. The same 10 individuals were observed only once for each trial at each temperature range. During a trial, one tadpole was gently placed in the centre of the chamber (15, 20, 25, 30 and 35°C, respectively, for the five gradients) and allowed to freely move around for 15 min (900 s). The water temperature of each location selected by the tadpole was verified with a thermometer after each experiment. In practice, the resolution of thermal selection was limited to whether the tadpoles moved from the centre of the gradient to the warmer or cooler end of the gradient. This was quantified by dividing the chamber into three 22 cm sections, with the middle third representing the mean temperature range and the upper and lower thirds the warmer or cooler ranges. For example, with the 10–20°C gradient, the lower third represented temperatures below 13°C and the upper third temperatures above 17°C. Stage and mass were recorded after each experiment. To prevent any effects of the brief exposure to the variations in thermal conditions, tadpoles were returned to their tanks for a minimum of 48 h between trials.

### Burst speed

Burst speed at four experimental temperatures (15, 20, 25 and 30°C) was determined for another subset of 10 individuals from each acclimation group. As with the thermal selection experiments, each of 10 individuals was evaluated only once at each temperature. The same 66 cm chamber used for the thermal selection experiments was used, except a uniform temperature was maintained along its length. Two electrodes connected to a Grass Technologies (West Warwick, RI, USA) SD9 stimulator were secured side by side at one end of the track that was similarly filled 2 cm deep with water obtained from the aquarium associated with the tadpole being tested. When the water reached the desired temperature, the tadpole was placed into the chamber and manoeuvred so it was at the end of the track facing the centre, with its tail between the electrodes. Once the tadpole was stationary in this position, an electrical stimulus of 80 V was delivered for 12 ms to elicit a burst speed response. Tadpole location was transmitted every 100 ms to the LAD and recorded by Microsoft HyperTerminal software (Redmond, WA, USA). Burst speed was calculated by selecting the longest distance travelled in a 500 ms period. Tadpoles were observed closely during and after the trials, with no discernable adverse effects on development or behaviour compared with tadpoles in the same group not used for the experiment.

### Analysis

Experimental data were analysed with an ANOVA factorial design with Tukey's HSD for *post hoc* comparisons (KaleidaGraph version 4.5.2). The significance level for *post hoc* comparisons was *P* ≤ 0.05. We used χ^2^ tests to compare the number of surviving vs. deceased tadpoles for the 20, 25 and 30°C acclimation groups. Data are presented as the mean ± SEM unless otherwise indicated.

## Results

### Thermal effects on development

The 15°C acclimation group had advanced to an average of only two Gosner stages after 191 days at that temperature. The average mass was 0.58 ± 0.16 g and survivorship decreased (Table 1). Owing to the lack of development, a decision was made to treat them as if they were experiencing an over-wintering event. By using the average regional daily temperatures from the US National Weather Service (www.noaa.com), we determined that springtime air temperatures increase in the general area by a rate of 1°C every 5 days. Water temperatures were increased at this rate until reaching 25°C, because the 25°C acclimation group had the quickest developmental rate. After 50 days, 25°C was reached and 80% of the remaining tadpoles (*n* = 30) survived. After reaching 25°C, 96% of the remaining tadpoles from the original 15°C group (*n* = 24) survived to metamorphosis in 42 ± 2 days (range 32–68 days), with a mass of 4.83 ± 1.08 g. This range of values for time to metamorphosis overlapped with the time required by the 25°C group to reach metamorphosis (see below).

**Table 1:** The effects of thermal acclimation on survival to metamorphosis, mass at metamorphosis and time to metamorphosis in the 2005 experimental group

Temperature (°C)	Count	Survival (%)	Mass (g)	Time (days)
15	47	64	0.58 ± 0.16	(*191*)
15→25	30	80	1.10 ± 0.76	(*50*)
15 at 25	24	96	4.83 ± 1.08	42 (32–68)
20	50	82	4.92 ± 1.14	264 (184–344)**
25	48	94	4.40 ± 0.72	67 (56–87)
30	47	66^†^	3.90 ± 0.84	98 (79–130)**
35	46	0	—	—

Survival (evaluated by χ^2^ analysis of the number of survivors) was significantly lower in the 30°C acclimation group than in 25°C group (^†^), and none of the tadpoles in the 35°C acclimation group reached metamorphosis. Owing to the lack of development at 15°C (stage 28 after 191 days), rearing temperature was increased to 25°C over a 50 day period (15→25) to simulate an over-wintering event (days italicized). Asterisks (**) show a significantly longer time to metamorphosis in the 20 and 30°C acclimation groups relative to the 25°C acclimation group (*P* < 0.001). Values are given as the mean (range) number of days required to reach Gosner stage 42.

The groups acclimated at 20, 25 and 30°C successfully reached metamorphosis (Table 1). Survivorship, evaluated by χ^2^ analysis, was lower in the 30°C acclimation group (66%) relative to the 25°C group (94%, *P* = 0.01) but not the 20°C group (82%, *P* = 0.07). The 25°C group had the shortest time to metamorphosis (56–87 days, average 67 ± 1.2 days), which was significantly shorter than the 30°C group (79–130 days, average 98 ± 2 days; *P* < 0.001). The 20°C group required a significantly longer time to reach metamorphosis than either the 25 or 30°C group (184–344 days, average 264 ± 7 days; *P* < 0.001). The mass at metamorphosis was not significantly different among the acclimation groups.

Tadpoles in the 35°C group appeared lethargic and thin despite continuous food availability. To increase survivorship, temperature was reduced in 2°C increments every 15 days until reaching 30°C. Despite the decrease in temperature, none of the tadpoles initially exposed to 35°C reached metamorphosis (Gosner stage 42). The last tadpole survived 143 days post-hatch and reached Gosner stage 27, with a mass of 0.27 g. Owing to the failure to metamorphose, results from the 35°C acclimation group are not presented.

### Thermal preference

**Figure 1:**
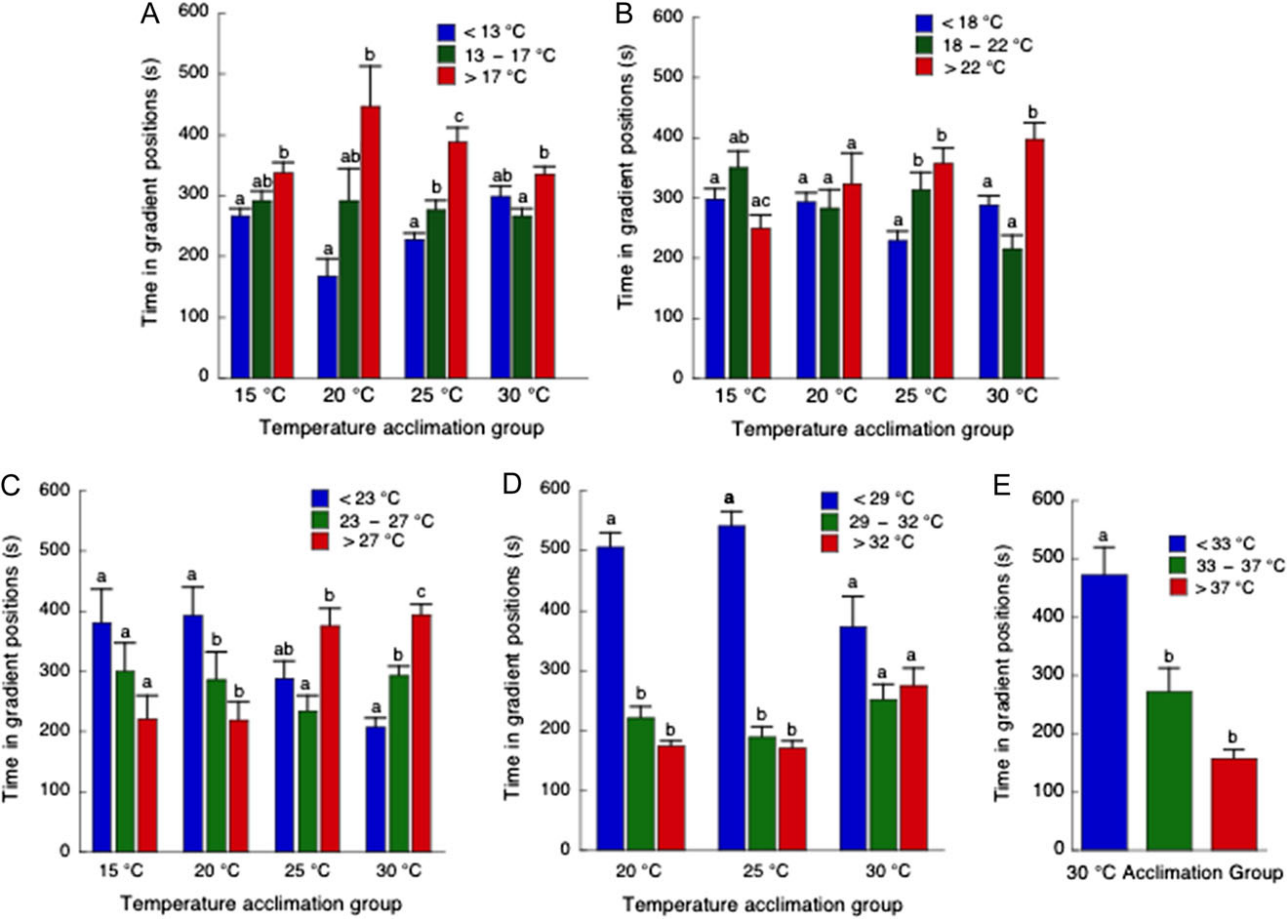
The effect of thermal acclimation on thermal preference in a 66 cm linear gradient with a 10°C temperature range. Five temperature ranges (**A**, 10–20°C; **B**, 15–25°C; **C**, 20–30°C; **D**, 25–35°C; and **E**, 30–40°C) were used to characterize the thermal preference of tadpoles acclimated to 15, 20, 25 and 30°C. Preference was evaluated for each acclimation group as the time spent in the cooler (blue), intermediate (green) or warmer thirds (red) of the gradient. See Table 2 for stage and mass of experimental groups. The bars represent the mean and one standard error for 10 individuals during a 900 s (15 min) trial. The same letters above the bars represent a lack of statistical difference (*P* > 0.05) between the time spent by a given acclimation group in each segment of a gradient. Fewer acclimation groups were used at the warmer gradients to minimize thermal effects on developmental progression.

Thermal preference data for each gradient were collected for tadpoles at different stages and body masses (Table 2). In the 10–20°C gradient (Fig. 1A), tadpoles from the 15 and 20°C acclimation groups selected warmer temperatures (>17°C) for longer periods of time than the cooler temperatures (<13°C; *P* = 0.001 and 0.004, respectively). The time spent in the middle (13–17°C) was intermediate and not significantly different from the warmer or cooler ranges of the gradient. Tadpoles from the 25°C acclimation group spent a significantly longer time at the warmer temperatures (>17°C) than either the middle and cooler temperatures (*P* < 0.001), and the time spent at the middle temperature (13–17°C) was greater than that in the cooler temperature (<13°C; *P* = 0.016). The 30°C group showed reduced locomotor activity when they moved to the cooler range of the gradient (<13°C), resulting in a greater than expected time in this region. Nonetheless, the amount of time spent at the warmer temperature (>17°C) was significantly greater than at the middle temperature (13–17°C; *P* = 0.001).

**Table 2:** Mean mass and stage (±SEM; Gosner, 1960) of tadpoles during thermal preference experiments

Temperature gradient	Acclimation group							
	15°C		20°C		25°C		30°C	
	Stage	Mass (g)	Stage	Mass (g)	Stage	Mass (g)	Stage	Mass (g)
10–20°C(±SEM)	27(0.7)	0.55(0.17)	36(4.6)	3.55(1.78)	39(1.1)	3.14(0.74)	31(3.4)	1.65(1.33)
15–25°C(±SEM)	27(0.2)	0.88(0.34)	36(1.3)	3.47(0.46)	38(1.4)	3.82(0.44)	32(1.0)	51.91(0.50)
20–30°C(±SEM)	27(0.5)	0.52(0.15)	35(4.6)	3.51(0.73)	38(2.1)	3.48(1.41)	35(4.4)	2.82(1.87)
25–35°C(±SEM)			35(2.0)	2.76(0.56)	39(0.4)	3.33(0.40)	35(1.3)	3.44(0.50)
30–40°C(±SEM)							35(1.4)	2.42(0.50)

Stage and mass varied between acclimation groups and gradients because experiments were performed on tadpoles from a single egg mass, developing at different rates. The 15°C group developed very slowly, and experiments were performed before temperatures were increased to 25°C.

In the 15–25°C gradient (Fig. 1B), the 15 and 20°C acclimation groups showed no significant preference between the warmer and cooler ends of the gradient. In contrast, the 25°C acclimation group showed significant preferences for the middle (18–22°C; *P* = 0.04) and warmer (>22°C; *P* < 0.002) thirds of the gradient, compared with the cooler end of the gradient. The 30°C acclimation group showed significant preference for the warmer third of the gradient, relative to the middle (*P* < 0.001) and cooler ends (*P* = 0.02).

In the 20–30°C gradient (Fig. 1C), the 15 and 20°C acclimation groups appeared to remain in the cooler (<23°C) third, but this was only significant for the 20°C acclimation group (*P* < 0.003). The 25°C acclimation group showed equivalent amounts of time in the warmer and cooler ranges of the gradient, with a greater amount of time at the warmer temperatures (>27°C) than the middle temperatures (23–27°C, *P* < 0.002). Tadpoles from the 30°C group spent a significantly greater amount of time at the warmer (>27°C) than both the middle (*P* < 0.001) and cooler temperatures (*P* < 0.001).

For fear that exposure to the 25–35°C gradient might adversely affect the 15°C acclimation group, only the 20, 25 and 30°C acclimation groups were tested (Fig. 1D). Notably, the 20 and 25°C acclimation groups showed a strong preference for the cooler third of the gradient (<29°C; *P* < 0.001 for both), while the 30°C acclimation group showed only a small, but not significant, tendency to remain in the cooler third. Thermal preference for the 30°C acclimation group in the 30–40°C gradient (Fig. 1E) showed significant avoidance of the warmer (>37°C) and middle thirds (>33°C) of the gradient relative to the cooler third (*P* < 0.001 and 0.002, respectively).

### Burst speed

**Figure 2:**
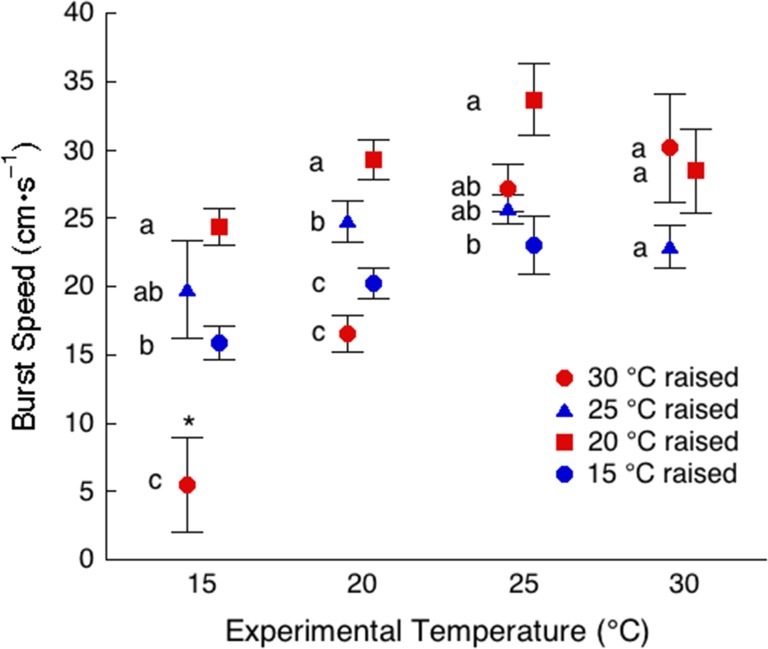
The effect of thermal acclimation on maximal burst speed at 15, 20, 25 and 30°C. Statistical comparisons are made between the acclimation groups within the same experimental temperature. Data points with the same letter are not significantly different (*P* > 0.05), whereas those with a different letter are significant (*P* ≤ 0.05). Each point represents a mean ± SEM (*n* = 10, except *n = 4).

Although there was a considerable amount of variation in body size within and between the tadpoles raised at the different temperatures (Table 3), we found surprisingly little variation in burst speed. Larger tadpoles had more mass to accelerate, which compensated for the shorter body length of the smaller tadpoles. For this reason, we present the results for burst speed as the absolute values (Fig. 2). The burst speed for the 15, 20 and 25°C groups showed a similar response to increasing experimental temperatures. The 25°C group had a greater burst speed than the 15°C group at experimental temperatures of 15, 20 and 25°C. The 15°C group was not tested at 30°C. The 20°C group was intermediate between the 15 and 25°C groups at experimental temperatures between 15 and 25°C. Of interest, the 30°C group had a significantly slower burst speed at 15°C than did the 15, 20 and 25°C groups. Burst speed for this group increased at an experimental temperature of 25°C to a value not different from the other groups. There was no statistical difference between 20, 25 and 30°C groups at an experimental temperature of 30°C.

**Table 3:** Mean stage and mass (±SEM) of tadpoles during maximal burst speed experiments

Acclimation group	Stage	Mass (g)	Range (g)
15°C	27.5 ± 0.6	0.51 ± 0.13	0.29–0.76
20°C	26.9 ± 0.8	0.26 ± 0.07	0.15–0.44
25°C	30.4 ± 2.4	0.84 ± 0.60	0.21–2.59
30°C	26.3 ± 0.8	0.13 ± 0.04	0.07–0.20

The large standard error for the 25°C acclimation group was attributable to their quicker development to metamorphosis.

## Discussion

### Thermal sensitivity for development

Survival of larvae to metamorphosis is a major contributor to amphibian reproductive success, as is the ability of newly metamorphosed young to reach reproductive age and continue their life cycle. Denver (1997) suggested that ‘…the timing of metamorphosis is the central characteristic in the life history of an amphibian’ and is influenced by a variety of biotic and abiotic factors. The longer it takes a tadpole to develop, the greater the chances of predation in the aquatic habitat (Crump, 1984). Conversely, longer developmental time may result in larger body size at metamorphosis, which can decrease risk of predation in the terrestrial habitat (Berven and Gill, 1983). Our experiments with *R. onca* larvae were conducted to control for all biotic and abiotic variables except temperature and showed 25°C to provide the best prospects for survival, rapid development and large body size at metamorphosis. The tadpoles reared at 30°C were not significantly smaller than the 25°C group, but developmental time was longer and survivorship lower. Tadpoles successfully metamorphosed at 20°C, but many required >8 months for completion of development. Tadpoles were not able to reach metamorphosis at 15°C, but survivors completed metamorphosis when the temperature was increased to 25°C. Thus, the warmer temperatures that result from seasonal warming and the ability to swim to warmer water near the spring source appear to be important for reproductive success and must be considered in efforts to reintroduce these frogs to habitats in their historical range.

Early stage tadpoles initially raised from 21 to 35°C rapidly became thin and were unable to progress through metamorphosis even when temperature was subsequently reduced to 25°C. These results differed from a 2004 preliminary study, where we obtained tadpoles that were reared at 21°C for 48 days post-hatch, vs. 10 days in the 2005 study. These tadpoles had progressed variably to Gosner stages 25–32, were able to withstand 14 days at 35°C, and 94% survived to metamorphosis when the temperature was subsequently reduced to 30°C. The thermal history of early development can affect subsequent thermal tolerance, as has previously been shown for *R. pipiens* from Alabama (Noland and Ultsch, 1981). The degree to which variability in the thermal sensitivity of development through metamorphosis results from genetic limitations vs. phenotypic plasticity (Newman, 1992) remains an important question, especially with increasing temperatures and changes in rainfall patterns associated with global climate change.

### Thermal selection

Our experiments were conducted as the tadpoles progressed through variable stages and increasing body mass. Lucas and Reynolds (1967) observed *R. pipiens* tadpoles to select warmer temperatures at later stages of metamorphosis and suggested, as did Noland and Ultsch (1981), that selection of warmer temperatures served to accelerate metamorphosis. The gradient used by Lucas and Reynolds (1967) was 140 cm in length and maintained a temperature range of 10–40°C. They found that thermal preference for a given range of developmental stages covered approximately a 20°C span, with the modal value serving as a reference for comparison between stages. The heating and cooling capacity and the length of our gradient limited the temperature range to 10°C, and there was mixing of the gradient when a tadpole was present. To compensate for these limitations, we conducted experiments with five 10°C gradients to provide better resolution of the effect of thermal acclimation on thermal selection.

Tadpoles from all acclimation groups, except the 30°C group, spent more time at the warmer range of the 10–20°C, which is consistent with the requirement that warmer temperatures are required for metamorphosis. Notably, tadpoles from the 30°C group also spent significantly more time in the cooler end of this gradient because they appeared to lose locomotor function at the lower temperature. Lucas and Reynolds (1967) made similar observations on *R. pipiens* with the longer gradient described above. The 15 and 20°C acclimation groups showed no preference between the warmer and cooler thirds of the 15–25°C gradient, whereas the 25 and 30°C acclimation groups spent significantly more time in the warmer (>22°C) third. In the 20–30°C gradient, tadpoles from the 15 and 20°C acclimation groups appeared to select the cooler (<23°C) third of the gradient, but this was significant only in the 20°C group. Conversely, the 25 and 30°C acclimation groups appeared to select the warmer third of the gradient (<27°C) but, this was significant only for the 30°C group, perhaps because of metabolic adjustments near the upper range of temperatures where metamorphosis can be successfully completed. Tadpoles from the 15°C acclimation group were not evaluated in the 25–35°C gradient because of possible long-term effects on developmental status. In this gradient, selection of the cooler (<29°C) third of the gradient become obvious and highly significant, while the 30°C acclimation group showed no significant preference within this temperature range. The 30°C acclimation group did show significantly more time in the cooler third (<33°C) of the 30–40°C gradient. In general, it appears that tadpoles acclimated to 15–25°C prefer temperatures >17°C and show little selectivity for temperatures between 18 and 27°C. As noted above, tadpoles acclimated to 30°C lose locomotor activity at temperatures below 13°C but show no significant preferences for temperature in the 25–35°C range. However, these tadpoles avoid temperatures >33°C when placed in a 30–40°C gradient. These results demonstrate that thermal history can affect thermal selection for warmer temperatures in ranges where development is most successful. In this regard, the temperature at the outflow of Bighorn Sheep Spring varies seasonally between 33.4 and 34.2°C (Moran *et al*., 2015).

### Burst speed

Tadpoles must avoid aquatic predators in order to reach metamorphosis successfully (Jennings, 1988; Jennings and Hayes, 1994). They are not known for long-distance endurance swimming. However, short bursts can propel them out of a predator's path (Chovanec, 1992). In this regard, we chose absolute speed rather than normalizing per body length. The most obvious difference in burst speed was by 30°C tadpoles at an experimental temperature of 15°C. The 30°C group was also the smallest and at the earliest stages, which could contribute to a slower burst speed. However, when the experimental temperature was increased to 25°C the speed of this group was not significantly slower than that of any of the other groups. The slowness of the 30°C group at 15°C is consistent with the reduced locomotor function seen with these tadpoles at cooler temperatures in the thermal preference experiments.

At experimental temperatures of 15, 20 and 25°C, the 25°C acclimation group was significantly faster than the 15°C group, which is consistent with observations that tadpoles of the 15°C group were less active and moved more slowly in their home aquarium. The 25°C group had the largest body mass of these three groups, which might have contributed to their faster speed. However, burst speed was not different between the 20, 25 and 30°C groups at 25°C despite differences in size and did not increase further at 30°C. Thus, burst speed within the optimal temperature range for metamorphosis would appear similar for tadpoles that are selecting temperatures between 20 and 30°C in their natural habitat.

### Summary and perspectives

We have shown in laboratory conditions that warmer habitat temperatures (~25°C) are important considerations for reintroduction of *R. onca* to its once native habitats. Our preliminary observations that survival at higher temperatures (e.g. 35°C) increased when tadpoles were allowed to develop to a later stage before exposure to the higher temperature suggest that the developmental stage is also an important factor in the establishment of reintroduction protocols. Warmer temperatures may also play a role in protection from infection by *Batrachiochytrium dendrobatidis*, which is causing widespread eradication of amphibian populations worldwide (Fisher *et al*., 2009). Evidence for this possibility is provided by Forrest and Schlaepfer (2011), who found that the prevalence of *B. dendrobatidis* infection of the closely related lowland leopard frog (*R. yavapaiensis*) declined with increasing native habitat water temperatures. Other factors, such as food availability, are also important. For example, Courtney Jones *et al*. (2015) recently found that stochastic food availability slowed the rate of metamorphosis by striped marsh frog (*Limnodynastes peroni*) tadpoles. Observations such as these, and our own, contribute to the understanding of the complexity of factors that contribute to successful reintroductions (Tasziz *et al*., 2014).
